# Dimensions and Interactions of Large T-Cell Surface Proteins

**DOI:** 10.3389/fimmu.2018.02215

**Published:** 2018-09-27

**Authors:** Victoria Junghans, Ana Mafalda Santos, Yuan Lui, Simon J. Davis, Peter Jönsson

**Affiliations:** ^1^Department of Chemistry, Lund University, Lund, Sweden; ^2^Weatherall Institute of Molecular Medicine, University of Oxford, Oxford, United Kingdom

**Keywords:** CD45, hydrodynamic trapping, glycoproteins, kinetic-segregation model, protein dimensions, protein interactions

## Abstract

The first step of the adaptive immune response involves the interaction of T cells that express T-cell receptors (TCRs) with peptide-loaded major histocompatibility complexes expressed by antigen-presenting cells (APCs). Exactly how this leads to activation of the TCR and to downstream signaling is uncertain, however. Recent findings suggest that one of the key events is the exclusion of the large receptor-type tyrosine phosphatase CD45, from close contacts formed at sites of T-cell/APC interaction. If this is true, a full understanding of how close contact formation leads to signaling would require insights into the structures of, and interactions between, large membrane proteins like CD45 and other proteins forming the glycocalyx, such as CD43. Structural insights into the overall dimensions of these proteins using crystallographic methods are hard to obtain, and their conformations on the cell surface are also unknown. Several imaging-based optical microscopy techniques have however been developed for analyzing protein dimensions and orientation on model cell surfaces with nanometer precision. Here we review some of these methods with a focus on the use of hydrodynamic trapping, which relies on liquid flow from a micropipette to move and trap membrane-associated fluorescently labeled molecules. Important insights that have been obtained include (i) how protein flexibility and coverage might affect the effective heights of these molecules, (ii) the height of proteins on the membrane as a key parameter determining how they will distribute in cell-cell contacts, and (iii) how repulsive interactions between the extracellular parts of the proteins influences protein aggregation and distribution.

## Introduction

**Figure 1:**
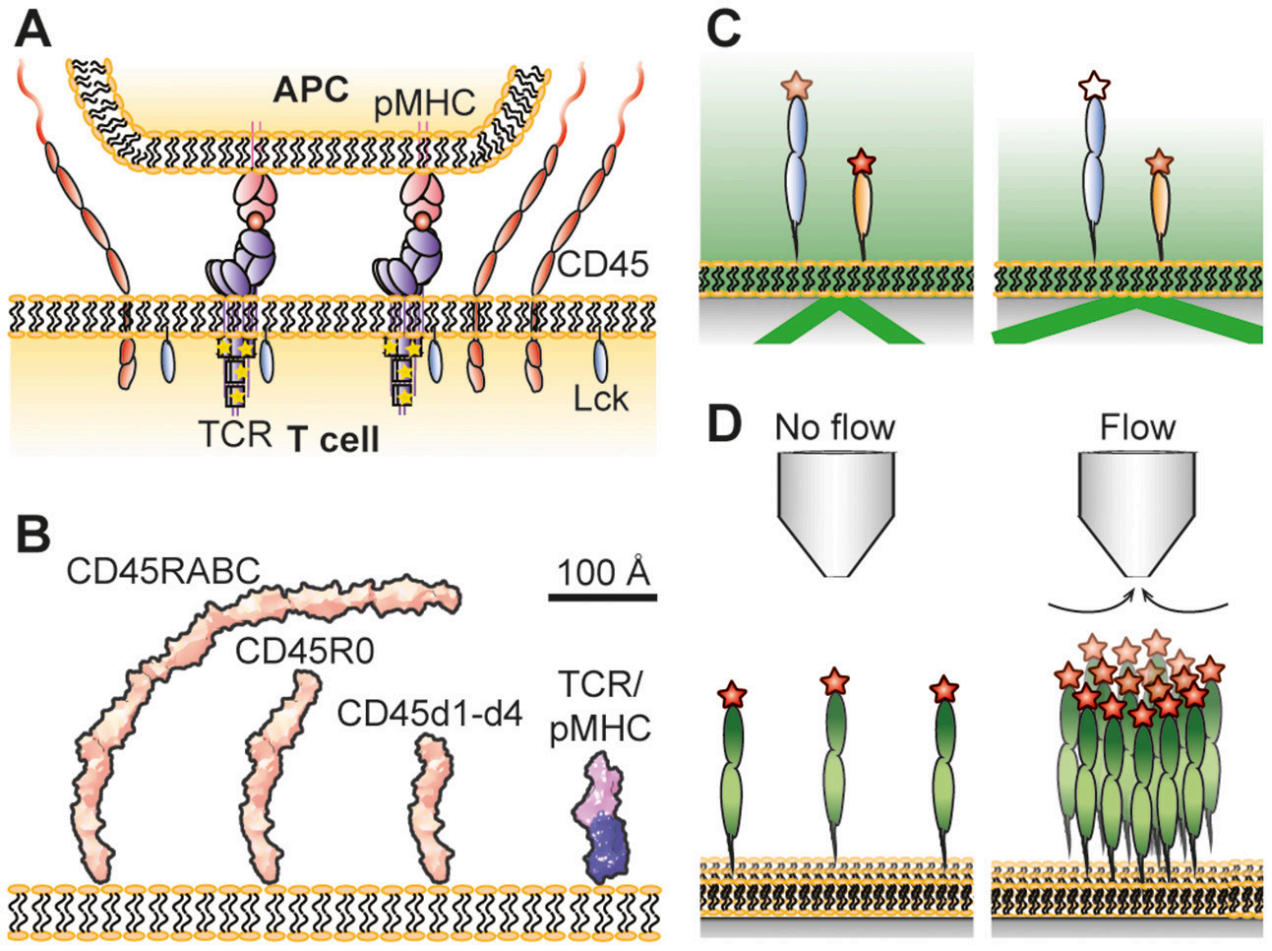
**(A)** Schematic illustrations of the kinetic-segregation model. CD45 keeps TCR unphosphorylated but gets excluded when TCR binds pMHC. This leads to TCR phosphorylation by Lck and T-cell signaling if the TCR stays long enough in the contact. **(B)** Dimensions of CD45 and the TCR/pMHC complex. The structures of CD45d1-d4 (8), and TCR/pMHC complexes (10, 11) have been determined by crystallography, and that of CD45R0 by negative-staining electron microsopy (8). CD45RABC is modeled on the CD45R0 structure, using the assumption that the mucin-like segment of RABC has the same length/mass ratio as in R0. The R0 and RABC forms of CD45 are each larger than the complex formed by the TCR and pMHC. **(C)** Principle of VA-TIRFM. Fluorophores at various heights will experience a different electromagnetic field when varying the incidence angle of the light, making it possible to determine their vertical position. **(D)** Hydrodynamic trapping uses the liquid flow from a micropipette to locally accumulate molecules. Measuring how much the molecules accumulate at different trap strengths makes it possible to determine the molecules' orientations and the intermolecular interactions between the molecules. Not drawn to scale.

The high specificity of the adaptive immune system is ensured by the interaction of molecular complexes on the surface of T lymphocytes called T-cell receptors (TCRs) with peptide-loaded major histocompatibility complexes (pMHCs) on antigen presenting cells (APCs). This interaction leads to activation of the TCR and further downstream signaling. However, how the TCR is triggered and initiates signaling is highly debated (1). Different mechanisms, including mechanotransduction (2–4) and receptor clustering (5, 6) to mention two examples, have been argued to play an important part in this. In addition, it has been observed that close contact zones form during the initial contact of T cells with model cell surfaces (7–9) and that the cell-surface bound phosphatase CD45 is partly excluded from these contacts, whereas shorter molecules such as the TCR and the protein kinase Lck are less affected (Figure 1A). This is in contrast to the formation of the supramolecular activation clusters that form minutes after T-cell activation (12, 13), whose organization is dependent on active transport by cytoskeletal motor proteins (14). The kinetic-segregation model has proposed that early, spontaneous molecular reorganization at close contact zones would lead to a local shift in the balance between phosphatases and kinases that results in the phosphorylation of the TCR complex and further downstream signaling if the TCR stays for a sufficiently long period in the CD45-depleted contact zone (15).

The possibility of sterically-based molecular segregation at cellular contacts poses the questions of how large proteins are organized and interact in general as well as the physicochemical mechanism driving segregation in these contacts. Various studies have been undertaken to understand this better including experiments using contacting cells (7, 16–18), immune cells interacting with functionalized surfaces (8, 19–21), and studies of fully artificial model systems (22, 23), to mention a few examples. It has long been argued that the key parameter that would drive segregation is the length of the protein compared to the cell-cell gap (15). However, whereas most of the cell-surface molecules involved in starting an immune response have been structurally characterized (24–26), the structures of the larger isoforms of CD45, as well as other bulky glycoproteins such as CD43, are less defined. Here we review several of the imaging-based optical microscopy techniques that have been developed for analyzing protein dimensions and orientation on model cell surfaces. We focus especially on the use of hydrodynamic trapping, which relies on liquid flow from a micropipette to manipulate membrane-associated molecules. Whereas values for the height and interactions between T-cell proteins would be needed to better understand the principles of the kinetic-segregation model, they will also be important to understand how proteins distribute on cell surfaces and in cell-cell contacts in general.

## Structural studies of CD43 and CD45

The T-cell glycocalyx consists of mainly CD43 and the receptor-type protein tyrosine phosphatase (RPTP) CD45, which are two heavily glycosylated glycoproteins. CD43 is comprised of a mucin-like extracellular region only, whereas CD45 has a folded region as well as variable N-terminal stretches of mucin-like sequence, with mucins being unfolded and extended, serine-, threonine-, and proline-rich polypeptide segments bearing large numbers of O-linked oligosaccharides (27, 28). The particular isoforms of CD45 that are expressed depend on cell type, developmental stage and cell activation state (29). These isoforms arise due to alternative splicing, which alters the length of the mucin-like region, with the smallest (CD45R0) and longest (CD45RABC) having mucin-like regions of 41 and 202 residues, respectively.

The crystal structure of the folded part of CD45 has been determined and comprises a beads-on-a-string arrangement of four fibronectin-like (FN3) domains (8). The organization and dimensions of the FN3 domains of CD45 are comparable to those in RPTPμ (30), another large type II RPTP, but the domain interfaces are larger, suggesting that flexibility in the extracellular region of CD45 is unhelpful for the function of CD45. Also, the topologies of the two N-terminal-most FN3 domains of CD45 are degenerate, i.e., some of the β-strands are absent or severely truncated, implying that the detail of the structure is unimportant. For this class of proteins, wherein levels of conservation can be as high as >90%, the sequence conservation of CD45 is remarkably low (~18% among mammalian species). The little conservation that there is, is mostly concentrated at the domain interfaces where it likely serves to influence the shape and rigidity of the extracellular domain of CD45, although additional conservation at the top of the folded part might impact on the positioning of the mucin-like region of the protein. The extracellular region of CD45 is also notable for being rich in cysteines that likely stabilize the protein *via* the formation of disulfide bonds; other large type II RPTPs either completely lack, or contain many fewer cysteines. Together, these observations point to the mechanical properties of the folded part of the extracellular domain of CD45 being integral to its function, as would be expected if it acts as a “lever” that excludes the attached cytoplasmic phosphatase from cell-cell contacts.

Obtaining the overall dimensions of glycoproteins using crystallographic methods is often difficult due to the presence of large amounts of sugar-based side chains which tends to work against the formation of crystals. The height of the intact ectodomains of CD43 and CD45 have instead been estimated using electron microscopy. Whereas the single mucin-like extracellular region of CD43 has a length of 45 nm (31), the ~15 nm folded region of CD45 combines with the mucin-like segments to give extracellular regions spanning 22 nm (CD45R0) to 40 nm (CD45RABC) (8, 32, 33). Comparisons with TCR-ligand complexes (~15 nm) (10, 11) suggested that even the smallest CD45 isoform, CD45R0, would be sterically excluded from sites of TCR-ligand engagement if CD45 has an “upright” posture at the cell surface (Figure 1B).

## The physical properties of cell-surface proteins measured *in situ*

Experiments have indicated that rigid membrane-anchored molecules can have markedly different surface heights due to their orientation, covering the range from being upright to lying flat on the surface depending on the conditions and their molecular properties (34–36). Rotation around the membrane-anchoring point can furthermore result in a significant change in effective height (34, 37). Several imaging techniques have been used to measure the effective height of membrane-anchored proteins and the gap size between contacting membranes. We will in this review focus on different optical methods. An alternative to these methods, not discussed here, is electron microscopy coupled with immunogold labeling, as implemented by Davis and colleagues, for example (38).

Reflection interference contrast microscopy (RICM) measures the gap size between contacting membranes by utilizing the interference pattern arising from light rays being reflected at different sample interfaces (39). From this information it has been possible to measure the gap size in artificial cell-cell contacts with nanometer precision (23, 40). The gap size in these studies was defined by the dimensions of binding receptor/ligand pairs bridging the two contacting surfaces. Another way to measure the length of receptor/ligand pairs is the microbead-based fluorescence imaging assay developed by Biswas and Groves (41). However, to measure the height of individual membrane proteins requires other methods. Fluorescence interference contrast microscopy (FLIC) (34, 42), scanning angle interference microscopy (SAIM) (43, 44) and variable angle total internal reflection fluorescence microscopy (VA-TIRFM) (8, 45) have all been used for this purpose. FLIC and SAIM are fluorescence-based interference methods, where the interference pattern arises due to different optical path lengths between the fluorophore and a reflective surface. For FLIC this is typically achieved by having oxide terraces of varying thickness between a reflective silicon wafer and the studied sample (42). The interference pattern will be different for each terrace which can be used to fit the effective height of the fluorophore above the surface. This has, for example, been used to measure the effective height of membrane-anchored glycopolymers (34) and reconstituted membrane proteins (36). SAIM creates the altering interference pattern by varying the incidence angle of the light. This makes it possible to make measurements on more heterogeneous samples compared to FLIC (46). Carbone et al. used this property of SAIM to measure the difference in height inside and outside artificial cell-cell contacts formed *via* a receptor/ligand pair, where the latter were enriched with CD45R0 (22). From this it could be concluded that membrane-anchored CD45R0 stands upright with an effective height similar to the full length of the protein. VA-TIRFM is a non-interferometric technique in which the sample is illuminated with light at an angle that is above that needed for total internal reflection (Figure 1C). This creates an evanescent electromagnetic field that decays exponentially with distance from the surface/sample interface (47). The decay length of the light depends on the incidence angle of the incoming light, and from the resulting change in emitted intensity from a fluorophore it is possible to estimate the vertical distance between the fluorophore and the surface (8). The relative heights of different CD45 isoforms anchored to a supported lipid bilayer have been studied using this technique (8). Both CD45R0 and CD45RABC had a significantly larger height than the folded, mucin-free region (i.e., FN3 domains 1–4 only). Unexpectedly, however, the apparent difference in height between CD45R0 and CD45RABC was only moderate. From these results it appears that the difference in effective height of membrane-anchored CD45RABC and CD45R0 could be much smaller than expected from the lengths of the proteins. A possible explanation for this, supported by hydrodynamic trapping studies, is that the larger protein can rotate around its anchoring point, leading to a lower effective height.

There are also other fluorescence-based methods that can be used to measure molecular height and orientation. Förster resonance energy transfer has been used extensively to map <10 nm distances and conformational changes in macromolecules (48). A related technique that extends the distance range to 100 nm, but retains the nm resolution, is metal-induced energy transfer (MIET) (49, 50). MIET measures the lifetime of fluorescent molecules above a metallized surface, which changes with the distance to the surface, and has a 3 nm resolution in living cells (50). The orientation of the membrane and membrane-associated molecules can also be estimated using polarization microscopy (51–53). This is based on the fact that the excitation and emission of fluorescent probes depends on the polarization of the incident light relative to the orientation of the fluorescent molecule. The average orientation of a labeled macromolecule can then be estimated if it is known how the fluorescent moiety orients relative to the labeled molecule (51, 52). In addition, there has been a rapid development of various superresolution fluorescence imaging techniques over the last decade with nm resolution along the optical axis (54). This includes, but is not limited to, biplane imaging (55), stochastic optical reconstruction microscopy with optical astigmatism (56), interferometric photoactivated localization microscopy (55), and 4Pi nanoscopy (57, 58). Protein heights can also be measured using two-color fluorescence imaging by relating the position of a dye on the end of the studied protein and another dye in the membrane or on the cytoplasmic part of the protein (18).

## Hydrodynamic trapping

### Measuring the height of membrane-anchored molecules

Hydrodynamic trapping uses the focused liquid flow from a micropipette to move and accumulate membrane-anchored molecules (59). It is the drag force on the molecules due to the flow that causes them to move in the direction of flow; larger molecules experience a higher drag force than smaller molecules (60). A micropipette is first positioned above a supported lipid bilayer (SLB) anchoring the studied proteins. After applying negative pressure over the micropipette, the proteins start to accumulate until a steady state concentration profile is reached (Figure 1D). During this process, the surface coverage can be increased by several orders of magnitude (59, 61). The steady state accumulation depends on the dimensions of the protein, with a protein standing upright accumulating more than a protein positioned at an angle (60). The accumulation will also be affected by intermolecular interactions among the proteins. By relating the amount of accumulation to the trap strength at different positions it is possible to determine both the orientation as well as the intermolecular interactions from a single measurement (62).

**Figure 2:**
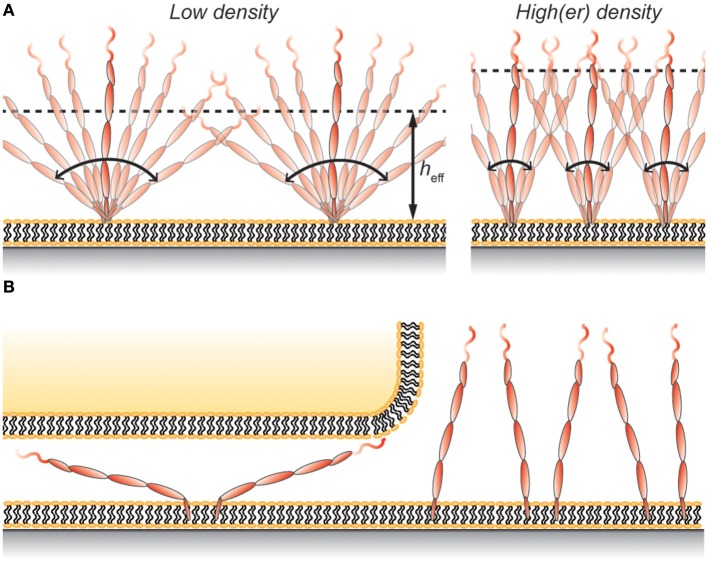
**(A)** Protein rotation gives rise to an effective height, *h*_eff_, that is lower than the full length of the protein. The value of *h*_eff_ depends, in addition to the properties of the protein, also on the protein density on the membrane. **(B)** Rotating membrane-anchored proteins can fit in gaps smaller than the length of the protein even without deforming the membrane. However, this restricts the possible angles the protein can have and comes at a cost in entropy, which leads to protein exclusion at the contact.

Using hydrodynamic trapping we found that the largest CD45 isoform, membrane-anchored CD45RABC, interacts with other CD45RABC molecules as if the protein was a 40 nm long rod that is free to rotate around its anchoring point (62). This means that the average height of the protein on the surface would be ~25 nm at low protein densities (Figure 2A). This is similar to the height of membrane-anchored CD45R0 measured by Carbone et al. (22), and can explain why Chang et al. (8) only found a modest difference in height between membrane-anchored CD45R0 and CD45RABC. However, it should be noted that the concentration of both CD43 and CD45 in the cell's glycocalyx is high, ~1,000 molecules/μm^2^ each (8, 63), and so interactions with neighboring proteins will cause CD45 to adopt a more upright position on the cell surface (Figure 2A). Indeed, using Monte Carlo simulations, similar to those described in Junghans et al. (62), we found that this effect would increase the effective height of CD45 by ~5 nm. Thus, the effective height depends not only on the total length of the protein, but also on other properties including flexibility and protein density.

It should be mentioned that only the interaction between the extracellular region of the proteins was studied and that the transmembrane domain of the proteins was replaced with a histidine tag that binds to nickel-chelating lipids in the SLB (62). Whereas this affects the mobility of the proteins compared to their native state on the cell surface, it should only moderately influence the steady state distribution in the hydrodynamic trap. However, it remains to be investigated whether the orientation and interaction of the proteins are affected by the mode of membrane attachment. This could, for example, be studied by comparing height measurements of the proteins on cells, or by using different model systems incorporating the full protein in a cushioned supported lipid bilayer (64).

### Measuring intermolecular interactions

How the effective height of surface proteins affects protein distribution in cell-cell contacts will be discussed in the next section. However, protein exclusion from cell-cell contacts is also influenced by crowding effects, and it has been observed that proteins smaller than the gap size can also be excluded (16, 18, 23, 65, 66). This can be due to interactions with stationary adhesion molecules in the contact but also with mobile proteins on the contacting surface. This topic has recently been covered in detail (23), however, it should be noted that the more repulsive the interaction between the proteins, the more pronounced the effect of crowding will be. Measuring these interactions can be done using hydrodynamic trapping (62), which will also indicate under what conditions the membrane-anchored molecules will cluster.

Protein rotation and glycosylation can both significantly increase the repulsion between membrane-anchored proteins. In fact, the latter can dominate the interaction between the proteins even if the total molecular weight of the added sugars is only a fraction of the total weight (62). Protein rotation around the anchoring point will also increase repulsion due to more frequent collisions with other proteins (62). Another effect of protein rotation and glycosylation is that a larger attractive energy is required to bring the proteins together into clusters. For example, no clustering or aggregation of the extracellular part of CD45RABC was observed when accumulating the protein at a trapping strength of 9 *k*_B_*T* from a surface coverage of 1,000 molecules/μm^2^ (62). Most of this repulsive energy came from free rotation of the protein around the anchoring point. If the rotation can be restricted by other means, such as binding to a receptor on a contacting cell, the repulsive energy would be reduced, and the likelihood of aggregation increase. An example of this is the cadherin adhesion proteins where lateral clustering of the proteins in junctions is amplified by *trans* interactions across the contacting cells (67, 68).

## Protein distribution at cell-cell contacts and its dependence on height

Proteins larger than the cell-cell gap generally get excluded, but the extent of exclusion can vary significantly depending on the system and the number of adhesion molecules creating the contact (16, 23). In understanding this it must be considered that the cell membrane is not rigid and can therefore bend to encompass molecules larger than the average cell-cell gap. This leads to an increase in energy of the system which is balanced by the cost of excluding a protein from the cell-cell contact (16). The number of adhesion molecules bridging the gap also influences the exclusion. For a high density of adhesion molecules (10,000 molecules/μm^2^) Schmid et al. found a relatively sharp transition between partially and fully excluded proteins, and that proteins 2 nm larger than the 10 nm gap were essentially completely excluded (23). Alakoskela et al. used a lower density of adhesion molecules (500 molecules/μm^2^) bridging two cell surfaces and forming a 15 nm cell-cell gap (16) and in this case the transition from no exclusion to exclusion was more gradual and even 25 nm diameter spherical particles were only excluded by 40–50%. This value is similar to the 60% exclusion of CD45R0 measured by Chang et al. in a 15 nm gap between a T cell and a supported lipid bilayer with a similar number of adhesion molecules (8). A slightly higher value for the exclusion in the study by Chang et al. agrees with the possibility that only the cell and not the supported lipid bilayer is able to deform and encompass the proteins. Thus, the similar effective height for the rod-like protein (8) and the spherical particle (16) gives comparable exclusion values, despite the different shapes. Having a mixture of adhesion molecules of different length can also influence exclusion. For example, only 10% exclusion of CD45R0 was observed by Cai et al. in a contact between a T cell and a functionalized supported lipid bilayer containing a mixture of 13 and 40 nm adhesion pairs (21).

Using a wholly non-cell based approach, Carbone et al. compared the exclusion of CD45R0 and CD45RABC in a 15 nm gap between a giant unilamellar vesicle and a supported lipid bilayer (22). In these experiments the exclusion of CD45R0 was ~20% whereas CD45RABC was excluded by 40–50% under the same conditions. The lower values compared to those stated by Chang et al. (8) could be due to the vesicle being more flexible than the T-cell membrane, or that the T-cell cytoskeleton exerts additional excluding forces on CD45. But if the effective heights of the two CD45 isoforms are similar, as implied by VA-TIRFM measurements (8), why was the CD45RABC isoform significantly more excluded in the experiments of Carbone et al.? A possible explanation is that the larger protein can rotate around its anchoring point resulting in a smaller effective height. One consequence of this is that the protein can fit into the gap even without deforming the membranes (Figure 2B). However, this comes with an entropic penalty since not all surface-to-protein angles will fit the gap. For example, excluding angles that makes the protein height larger than 25 nm (angles >39°), which is the approximate height of CD45R0 (22), gives an estimated CD45RABC exclusion that is (39/90°)^−1^ = 2.3 times larger than that of CD45R0. A more accurate analysis would have to take molecular interactions as well as additional membrane deformation into account, but this approximation still captures the essential behavior observed by Carbone et al. (22).

## Concluding remarks

We have discussed that it is not only important to know the molecular dimensions of membrane-anchored proteins to understand how they behave and distribute at cell-cell contacts but also how these proteins orient and interact on the membrane. Fluorescence-based techniques have been developed that allow the effective height of a specific membrane-anchored protein to be obtained, however, in most of the studies done to date the protein is anchored to a model membrane. These model systems have nevertheless shown that the actual height *in vivo* could be different, especially if the membrane protein composition and density vary, as discussed above. Hence, general methods to measure the height of membrane-anchored molecules on the surface of cells are needed. Super-resolution fluorescence imaging is one technique that has shown promising developments in recent years to achieve this. However, knowing both the effective height of the protein and its crystal structure are not sufficient to predict how the protein will distribute at cell-cell contacts. The interactions between proteins and whether they can rotate also affects this. Hydrodynamic trapping can be used to measure these interactions and combined with knowledge of the protein structure reveal the conditions required for the proteins to partition into domains or aggregate on the membrane. Taking all of this into consideration, complementary techniques are needed to understand the complex behavior of these molecules and how they distribute at cell-cell contacts.

## Author contributions

VJ, AS, SD, YL, and PJ wrote the paper. VJ, PJ, and YL made the figures.

### Conflict of interest statement

The authors declare that the research was conducted in the absence of any commercial or financial relationships that could be construed as a potential conflict of interest.
